# Supplementary figures and images for: Ribosomal Protein S4 X-Linked as a Novel Modulator of MDM2 Stability by Suppressing MDM2 Auto-Ubiquitination and SCF Complex-Mediated Ubiquitination
Source: Biomolecules. 2024 Jul 23;14(8):885. doi: 10.3390/biom14080885 (PMC11351588; doi:10.3390/biom14080885)

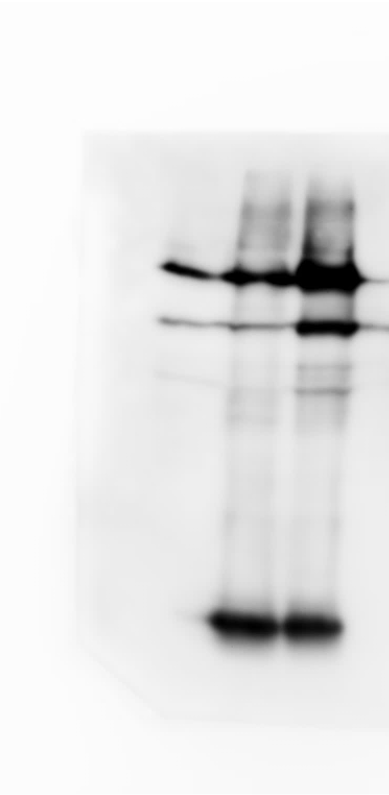

Supplement: Supplementary file 1 [file biomolecules-14-00885-s001.zip › Figure S1A. 1st panel.tif]

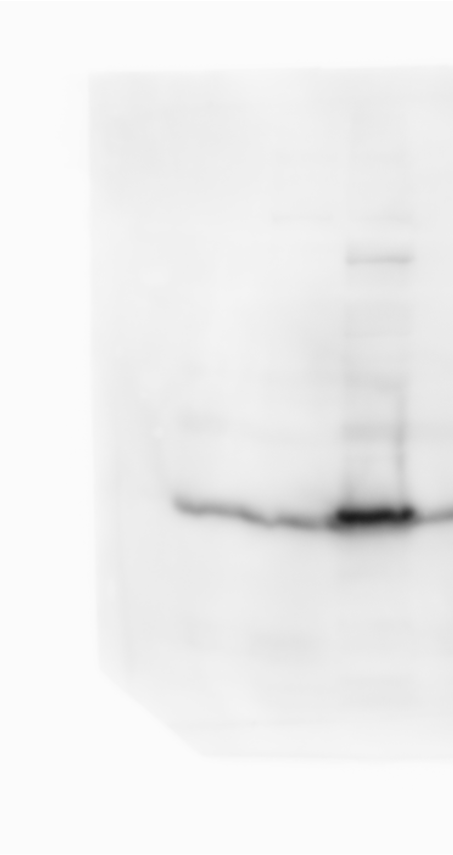

Supplement: Supplementary file 1 [file biomolecules-14-00885-s001.zip › Figure S1A. 2nd panel.tif]

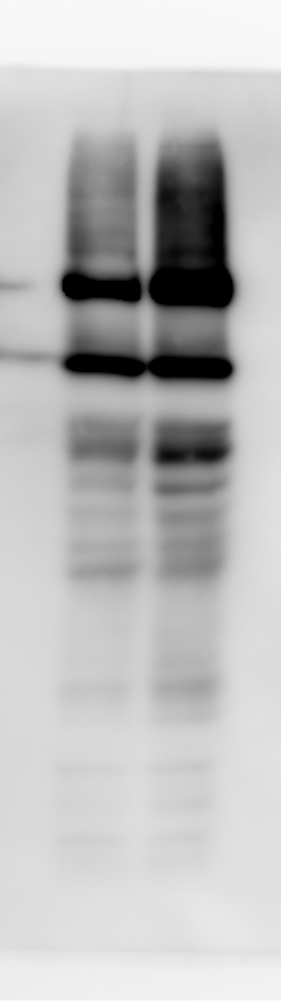

Supplement: Supplementary file 1 [file biomolecules-14-00885-s001.zip › Figure S1A. 3rd panel.tif]

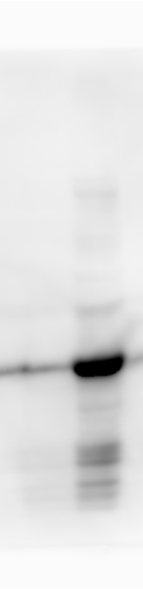

Supplement: Supplementary file 1 [file biomolecules-14-00885-s001.zip › Figure S1A. 4th panel.tif]

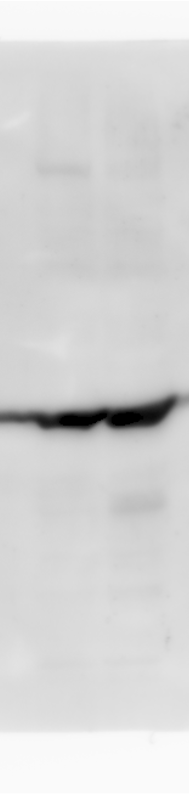

Supplement: Supplementary file 1 [file biomolecules-14-00885-s001.zip › Figure S1A. 5th panel.tif]

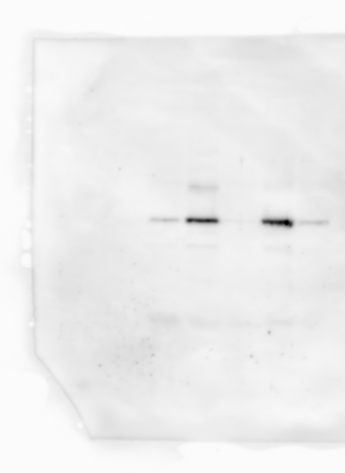

Supplement: Supplementary file 1 [file biomolecules-14-00885-s001.zip › Figure S2B. 1st panel.tif]

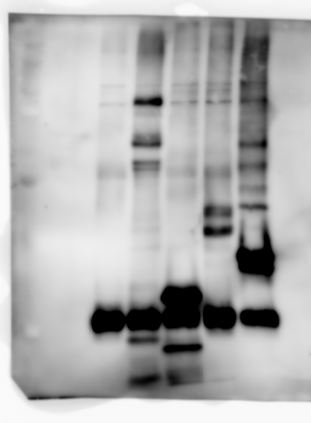

Supplement: Supplementary file 1 [file biomolecules-14-00885-s001.zip › Figure S2B. 2nd panel.tif]

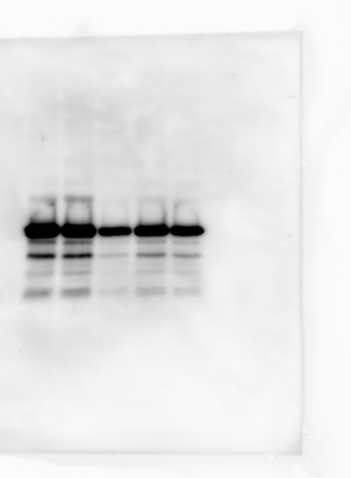

Supplement: Supplementary file 1 [file biomolecules-14-00885-s001.zip › Figure S2B. 3rd panel.tif]

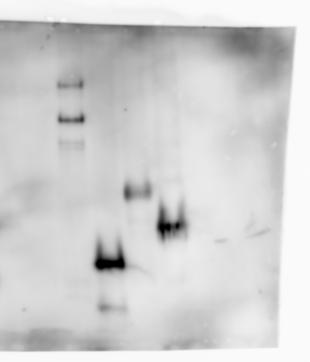

Supplement: Supplementary file 1 [file biomolecules-14-00885-s001.zip › Figure S2B. 4th panel.tif]

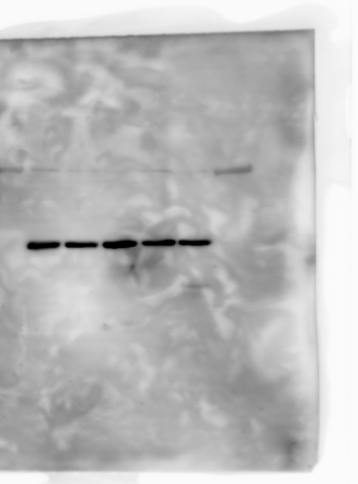

Supplement: Supplementary file 1 [file biomolecules-14-00885-s001.zip › Figure S2B. 5th panel.tif]

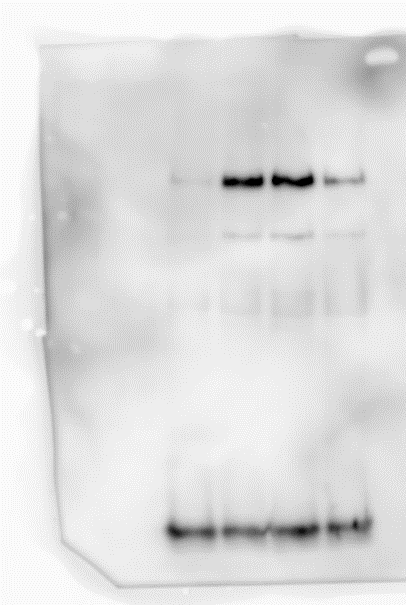

Supplement: Supplementary file 1 [file biomolecules-14-00885-s001.zip › Figure S2C. 1st panel.tif]

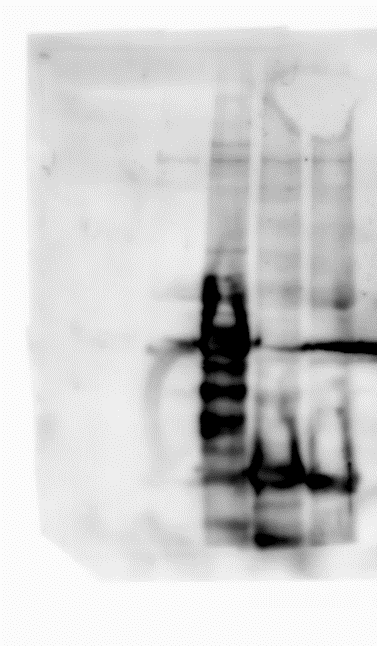

Supplement: Supplementary file 1 [file biomolecules-14-00885-s001.zip › Figure S2C. 2nd panel.tif]

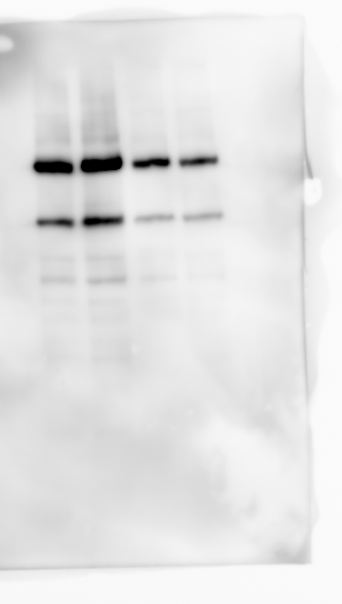

Supplement: Supplementary file 1 [file biomolecules-14-00885-s001.zip › Figure S2C. 3rd panel.tif]

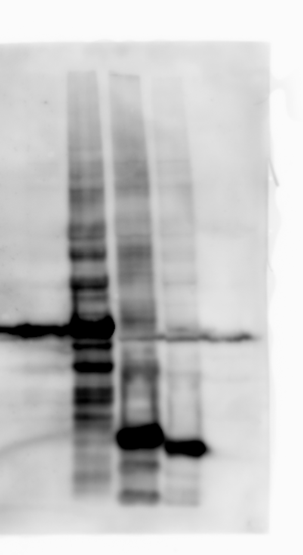

Supplement: Supplementary file 1 [file biomolecules-14-00885-s001.zip › Figure S2C. 4th panel.tif]

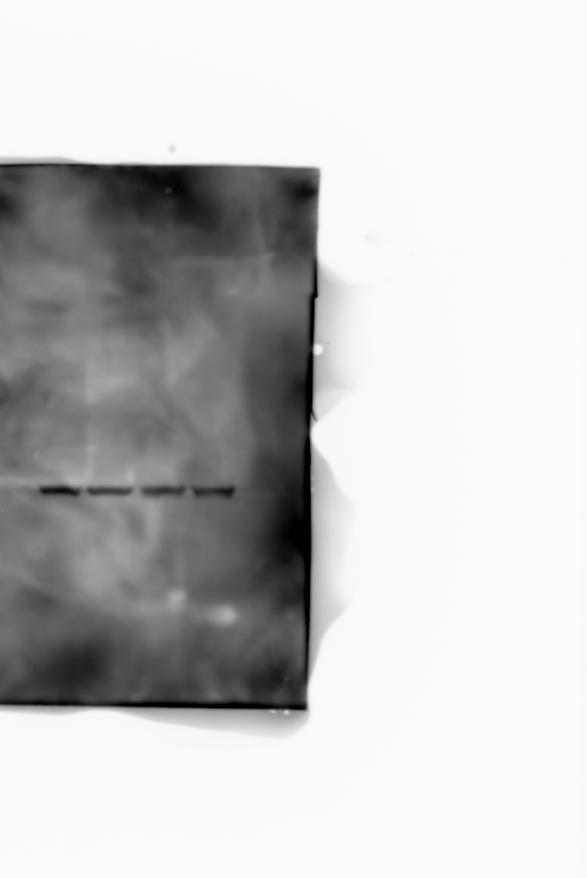

Supplement: Supplementary file 1 [file biomolecules-14-00885-s001.zip › Figure S2C. 5th panel.tif]

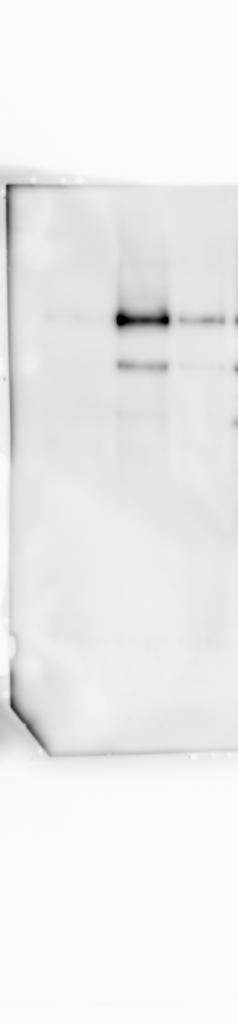

Supplement: Supplementary file 1 [file biomolecules-14-00885-s001.zip › Figure S3A. 1st panel.tif]

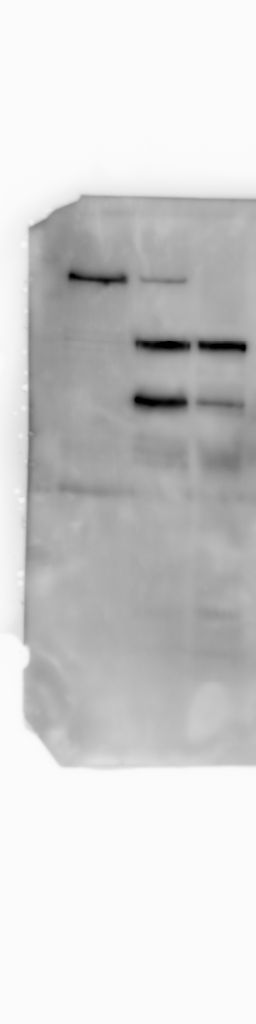

Supplement: Supplementary file 1 [file biomolecules-14-00885-s001.zip › Figure S3A. 2nd panel.tif]

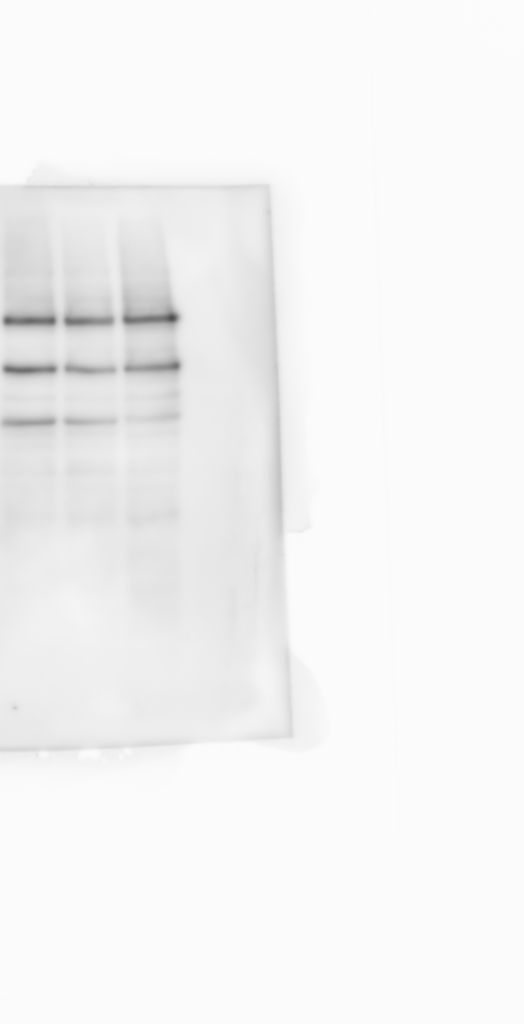

Supplement: Supplementary file 1 [file biomolecules-14-00885-s001.zip › Figure S3A. 3rd panel.tif]

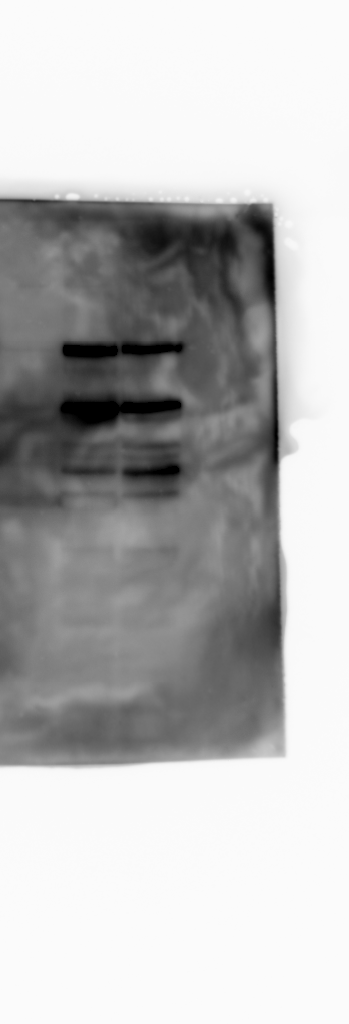

Supplement: Supplementary file 1 [file biomolecules-14-00885-s001.zip › Figure S3A. 4th panel.tif]

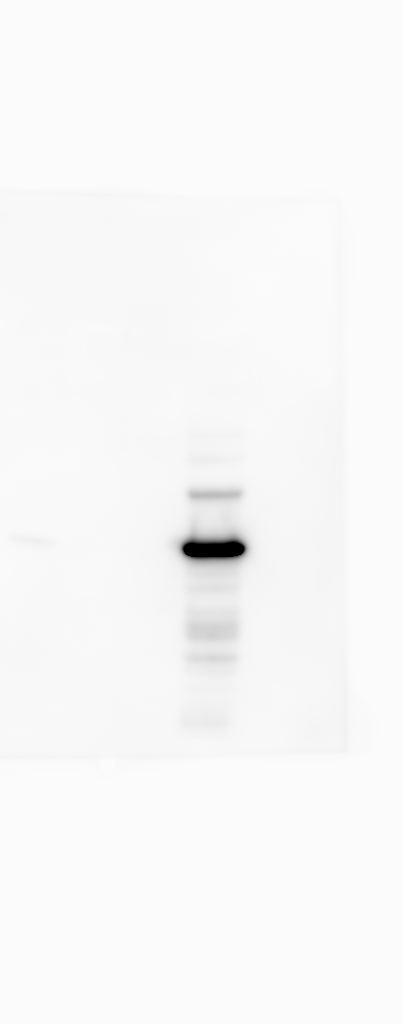

Supplement: Supplementary file 1 [file biomolecules-14-00885-s001.zip › Figure S3A. 5th panel.tif]

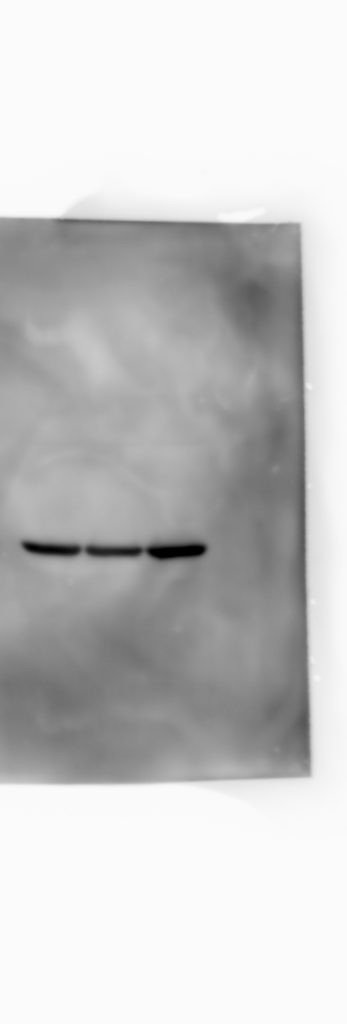

Supplement: Supplementary file 1 [file biomolecules-14-00885-s001.zip › Figure S3A. 6th panel.tif]

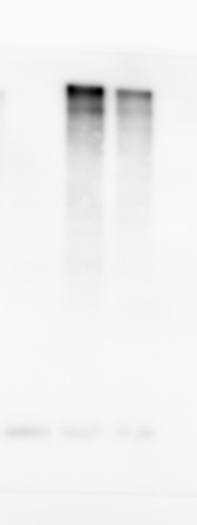

Supplement: Supplementary file 1 [file biomolecules-14-00885-s001.zip › Figure S3B. 1st panel.tif]

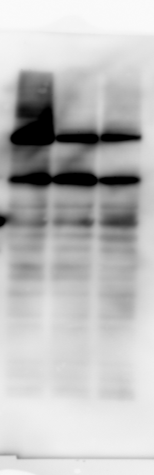

Supplement: Supplementary file 1 [file biomolecules-14-00885-s001.zip › Figure S3B. 2nd panel.tif]

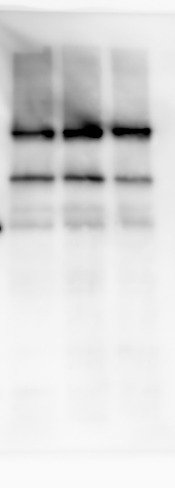

Supplement: Supplementary file 1 [file biomolecules-14-00885-s001.zip › Figure S3B. 3rd panel.tif]

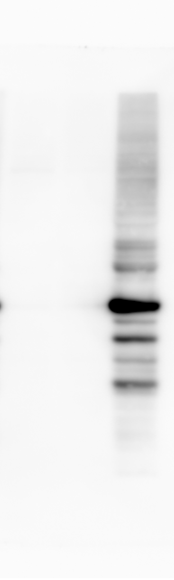

Supplement: Supplementary file 1 [file biomolecules-14-00885-s001.zip › Figure S3B. 4th panel.tif]

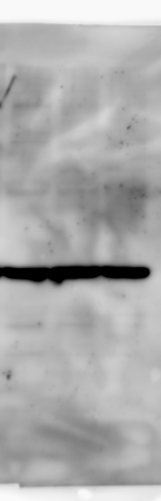

Supplement: Supplementary file 1 [file biomolecules-14-00885-s001.zip › Figure S3B. 5th panel.tif]

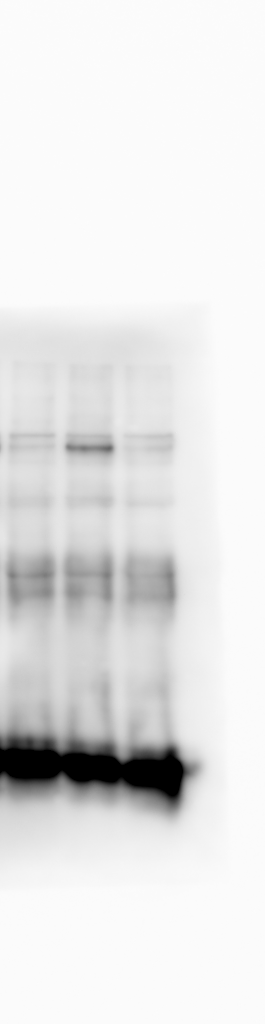

Supplement: Supplementary file 1 [file biomolecules-14-00885-s001.zip › Figure S4A. 1st panel.tif]

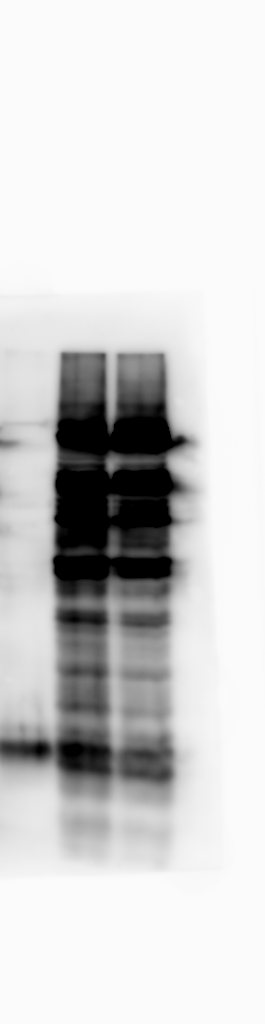

Supplement: Supplementary file 1 [file biomolecules-14-00885-s001.zip › Figure S4A. 2nd panel.tif]

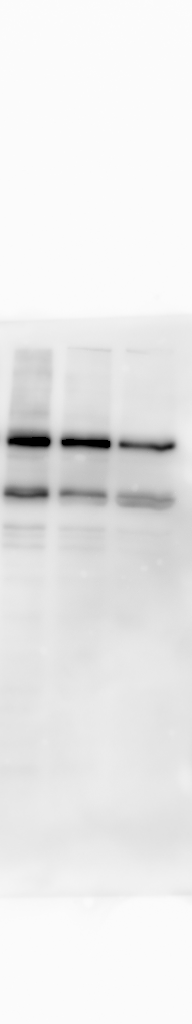

Supplement: Supplementary file 1 [file biomolecules-14-00885-s001.zip › Figure S4A. 3rd panel.tif]

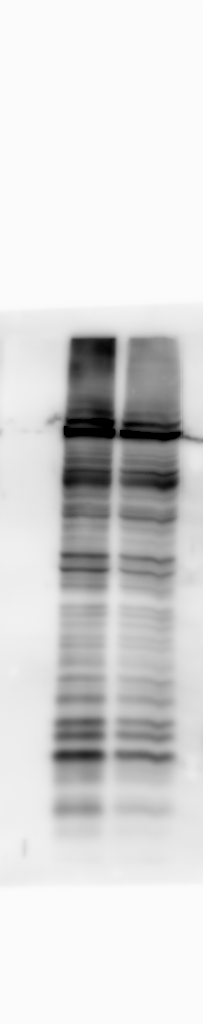

Supplement: Supplementary file 1 [file biomolecules-14-00885-s001.zip › Figure S4A. 4th panel.tif]

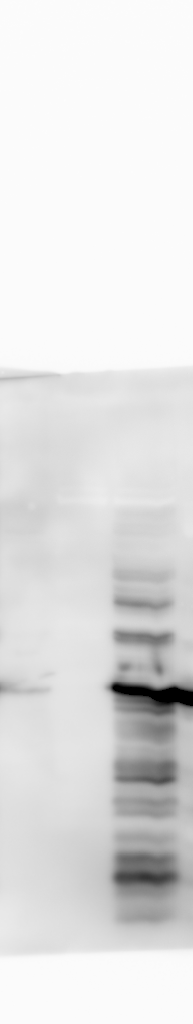

Supplement: Supplementary file 1 [file biomolecules-14-00885-s001.zip › Figure S4A. 5th panel.tif]

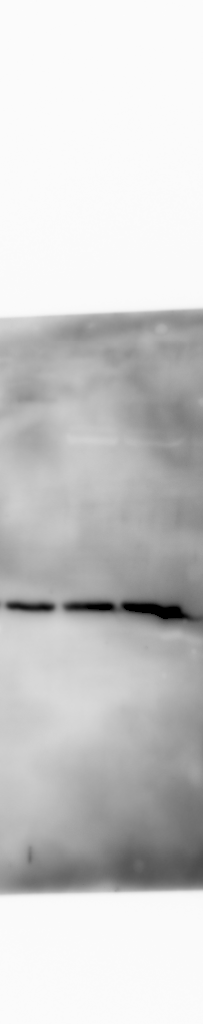

Supplement: Supplementary file 1 [file biomolecules-14-00885-s001.zip › Figure S4A. 6th panel.tif]

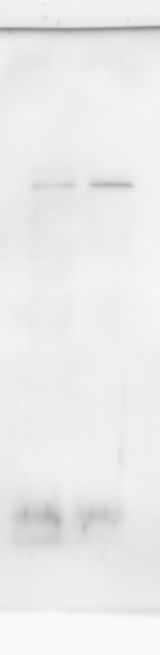

Supplement: Supplementary file 1 [file biomolecules-14-00885-s001.zip › Figure S4B. 1st panel.tif]

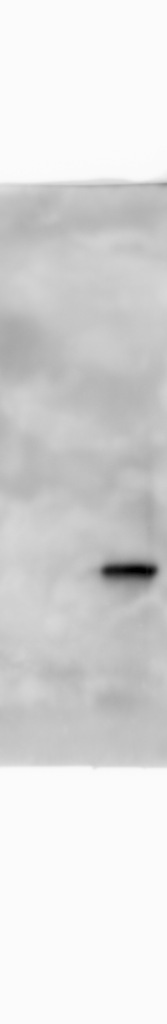

Supplement: Supplementary file 1 [file biomolecules-14-00885-s001.zip › Figure S4B. 2nd panel.tif]

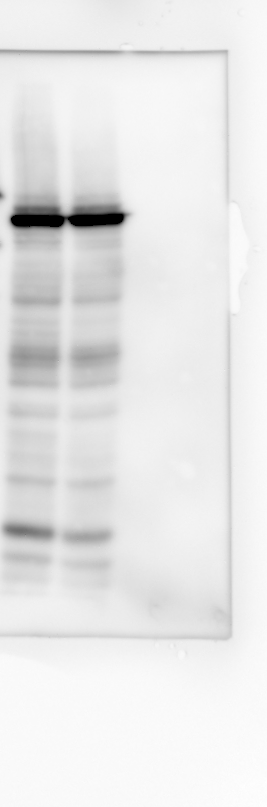

Supplement: Supplementary file 1 [file biomolecules-14-00885-s001.zip › Figure S4B. 3rd panel.tif]

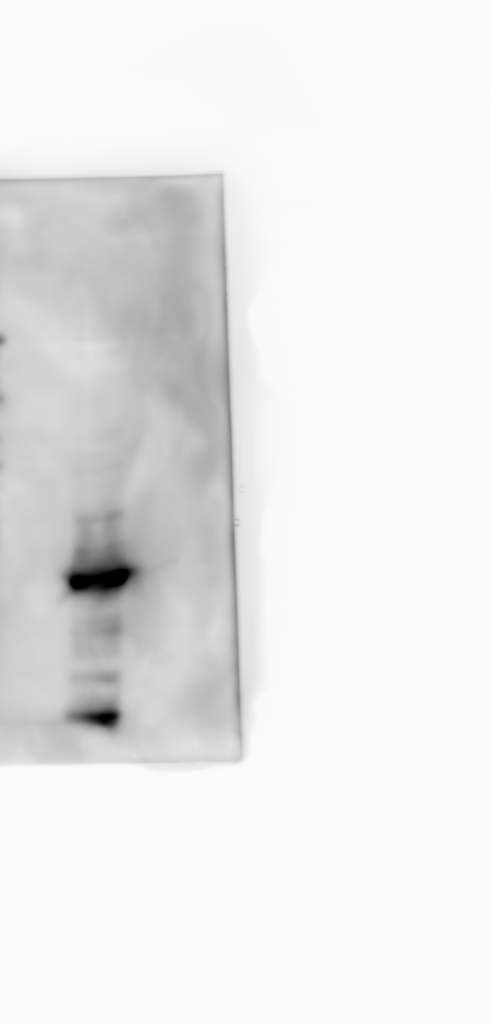

Supplement: Supplementary file 1 [file biomolecules-14-00885-s001.zip › Figure S4B. 4th panel.tif]

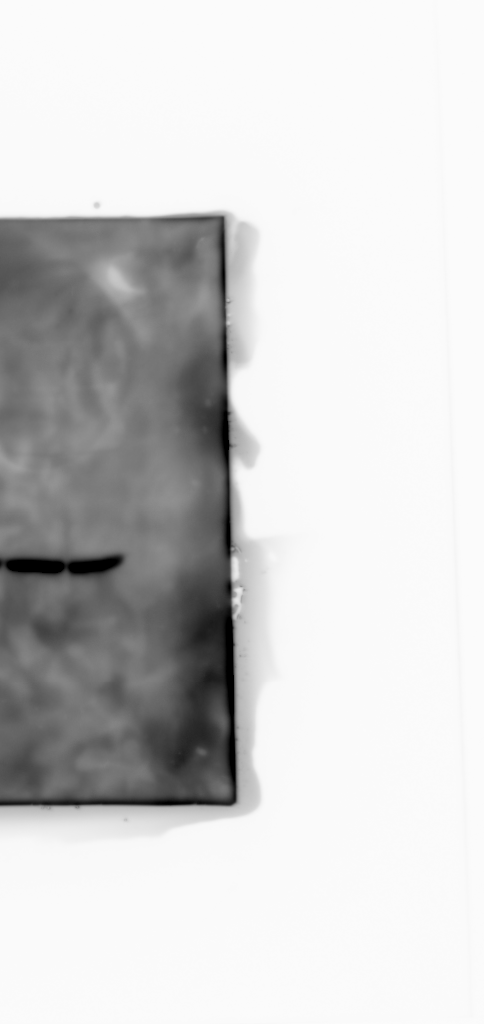

Supplement: Supplementary file 1 [file biomolecules-14-00885-s001.zip › Figure S4B. 5th panel.tif]

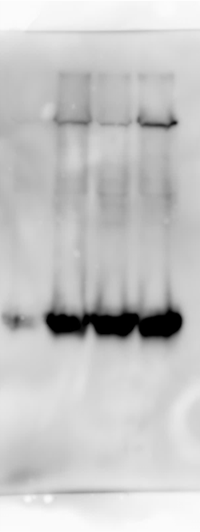

Supplement: Supplementary file 1 [file biomolecules-14-00885-s001.zip › Figure S4C. 1st panel.tif]

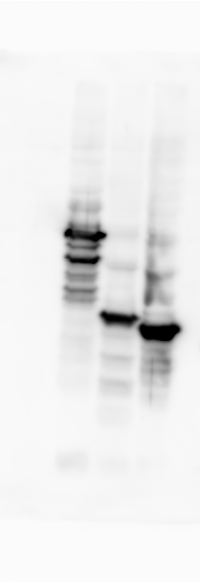

Supplement: Supplementary file 1 [file biomolecules-14-00885-s001.zip › Figure S4C. 2nd panel.tif]

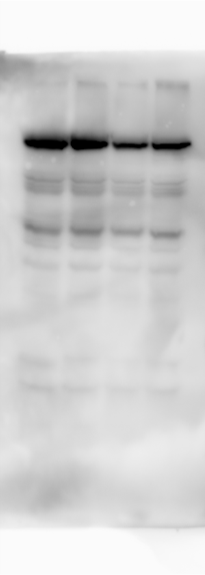

Supplement: Supplementary file 1 [file biomolecules-14-00885-s001.zip › Figure S4C. 3rd panel.tif]

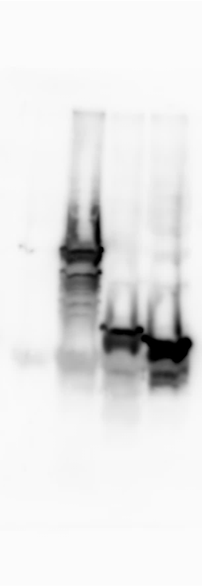

Supplement: Supplementary file 1 [file biomolecules-14-00885-s001.zip › Figure S4C. 4th panel.tif]

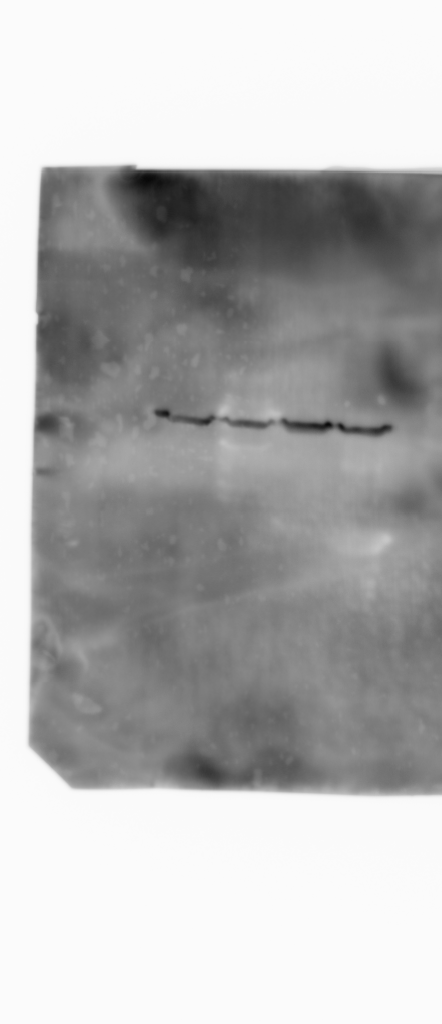

Supplement: Supplementary file 1 [file biomolecules-14-00885-s001.zip › Figure S4C. 5th panel.tif]

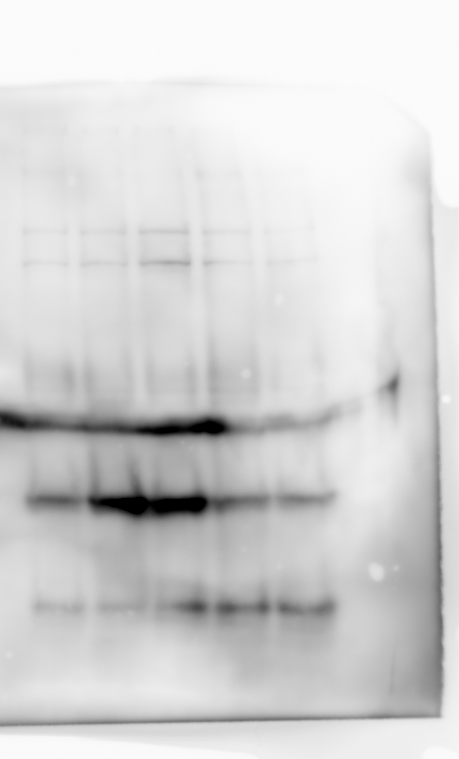

Supplement: Supplementary file 1 [file biomolecules-14-00885-s001.zip › Figure S4E. 1st panel.tif]

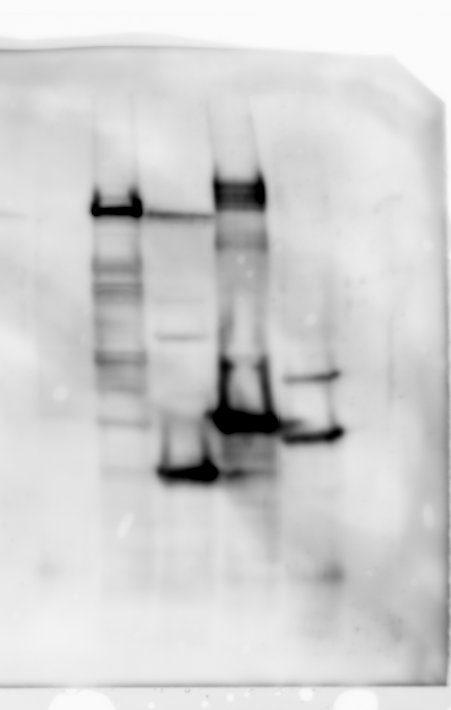

Supplement: Supplementary file 1 [file biomolecules-14-00885-s001.zip › Figure S4E. 2nd panel.tif]

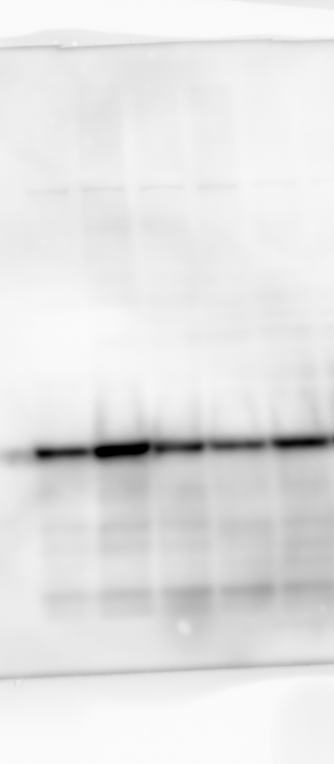

Supplement: Supplementary file 1 [file biomolecules-14-00885-s001.zip › Figure S4E. 3rd panel.tif]

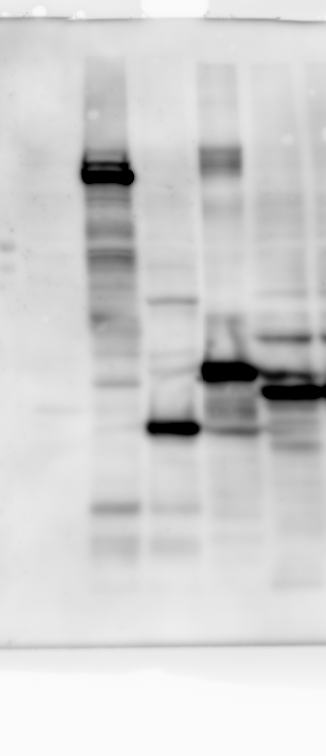

Supplement: Supplementary file 1 [file biomolecules-14-00885-s001.zip › Figure S4E. 4th panel.tif]

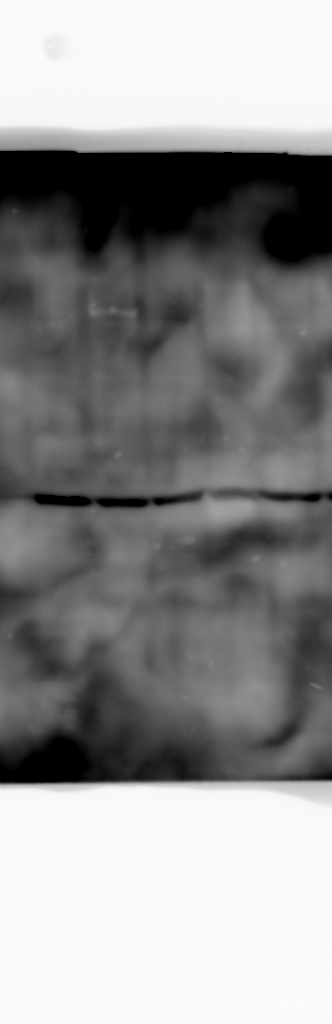

Supplement: Supplementary file 1 [file biomolecules-14-00885-s001.zip › Figure S4E. 5th panel.tif]

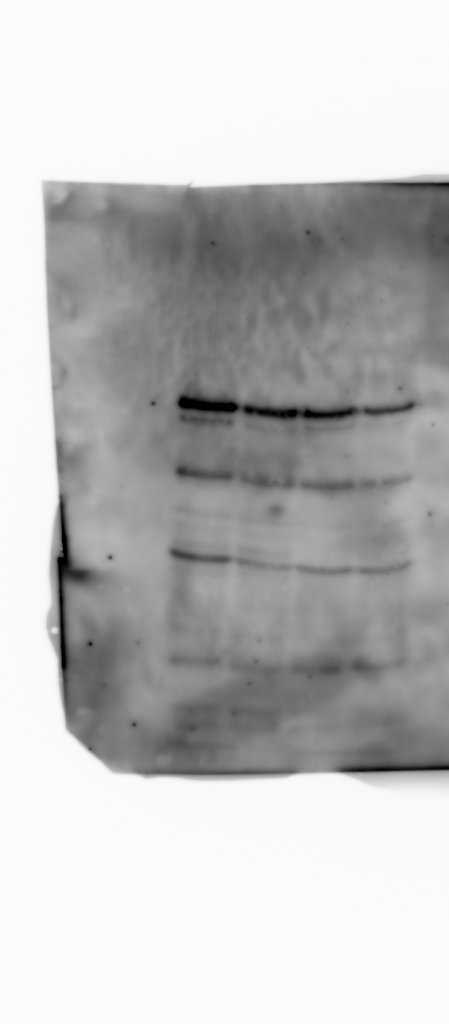

Supplement: Supplementary file 1 [file biomolecules-14-00885-s001.zip › Figure S5A. 1st panel.tif]

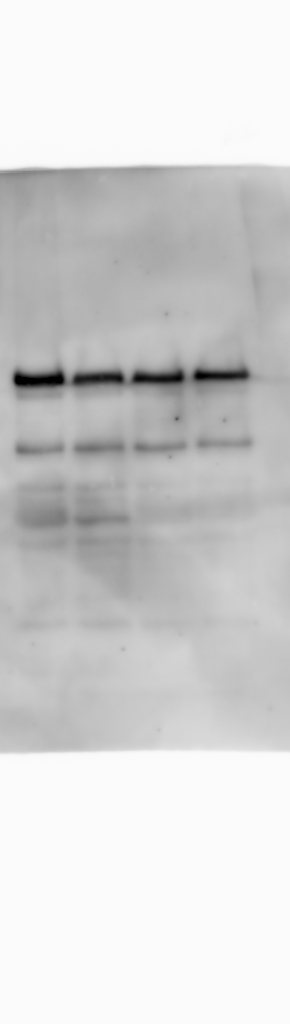

Supplement: Supplementary file 1 [file biomolecules-14-00885-s001.zip › Figure S5A. 2nd panel.tif]
